# Determinants of Quality Care and Mortality for Patients With Locally Advanced Cervical Cancer in Virginia

**DOI:** 10.1097/MD.0000000000002913

**Published:** 2016-03-03

**Authors:** Timothy N. Showalter, Fabian Camacho, Leigh A. Cantrell, Roger T. Anderson

**Affiliations:** From the Department of Radiation Oncology (TNS), Department of Public Health Sciences (FC, RTA) and Department of Obstetrics and Gynecology (LAC), Division of Gynecologic Oncology, University of Virginia School of Medicine, Charlottesville, VA.

## Abstract

Outcomes for patients with locally advanced cervical cancer are influenced by receipt of all indicated components of quality care: early diagnosis and receipt of external beam radiation therapy, chemotherapy, and brachytherapy. We performed an observational cohort study to evaluate receipt of quality cancer care and mortality after cancer diagnosis among patients with locally advanced cervical cancer in Virginia.

We queried the Virginia state cancer registry to identify patients with International Federation of Gynecology and Obstetrics Stage IB-IVA cervical cancer who were diagnosed during 2002 to 2012. We evaluated the influence of tumor-related, demographic, and geospatial factors on the receipt of indicated therapies and mortality. Treatment quality score of 0 to 3 was defined based upon the extent of receipt of the components of indicated therapy.

A total of 1048 patients were identified; 33.1% received all 3 components of treatment and only 54.0% received brachytherapy. Predictors of higher quality score included younger age group versus 66+ years at diagnosis (18–42 odds ratio [OR] = 12.3, 95% confidence interval: 6.6, 23.0; 42–53 OR = 5.6, CI: 3.3, 9.5; 53–66 OR = 5.5, CI: 3.3, 9.1), lower tumor stages versus IVA (IB2 OR = 3.3, CI: 1.8, 6.2; II OR = 2.7, CI: 1.6, 4.5; IIIx OR = 2.1, CI: 1.3, 3.6), and treatment at a high-volume facility (OR 2.2, CI: 1.2, 4.2). Predictors of increased mortality included earlier year of diagnosis, higher tumor stage, treatment at a lower volume facility, and lower treatment quality score.

In a cohort of locally advanced cervical cancer patients in Virginia, we identified a low rate of receipt of complete quality care for cervical cancer and a strong effect of facility volume on quality treatment and survival. Further research is needed to develop strategies to improve access to quality treatment and outcomes for cervical cancer.

## INTRODUCTION

Cervical cancer is the third most common gynecologic cancer in the United States, with an estimated 12,360 new diagnoses and 4020 deaths in 2014.^1^ Although cervical cancer rates in the United States have declined in recent decades, women in poor and rural communities and Appalachian states in particular are less likely to receive cervical cancer screening and have a higher incidence of cervical cancer and mortality.^2,3^ Disparities in cervical cancer incidence and cervical cancer-related mortality have been identified throughout the Appalachian region.^3^

Although early stage cervical cancer can be treated with surgery alone, treatment for locally advanced cervical cancer is complex and requires coordination of concurrent chemotherapy (CT), external beam radiation therapy (EBRT), and brachytherapy (BT).^4^ Delivery of BT requires technical proficiency with additional resources and expertise beyond that required for EBRT. Furthermore, the addition of concurrent CT to radiation therapy increases the risks of treatment-related adverse effects,^5^ requiring coordinated supportive care to avoid treatment delays and hospitalizations during the treatment course. Overall treatment duration has also been shown to affect outcomes, and delays reduce local tumor control and survival. Therefore, guidelines state that the chemo-radiotherapy course must be completed within 56 days,^6^ which demands coordination among health care providers.

Recent literature suggests low and decreasing compliance with quality care for cervical cancer, including BT and CT, at the national level.^7^ Although most women treated at academic medical centers receive cervical cancer treatment that meets basic quality standards, only 36% to 43% of women treated at community facilities receive standard of care treatments. For example, women treated at nonacademic community facilities are more than twice as likely to receive treatment without BT and receive CT at a lower rate.^4^ Potential explanations have been proposed, including declining expertise in BT, since radiation oncology resident physicians now frequently report limited exposure to cervical cancer BT during their training, as well as decreasing availability of BT services in radiation oncology centers.^8^

In Appalachian states, academic and/or large-volume medical centers are concentrated near medium to large metropolitan areas, making access to high-quality cancer care especially challenging for poor and uninsured patients who live a long distance away in rural or underserved communities. We therefore performed an observational cohort study to evaluate how receipt of quality definitive treatment of locally advanced cervical cancer in Virginia is influenced by disparities in access to high-volume and/or academic cancer centers, health insurance status, poverty, and rural environment. We also evaluated the associations between receipt of quality cervical cancer treatment and survival. This study adds to the existing literature by focusing on a modern cohort, in era where contemporary treatment methods were already established, that includes a population with rural risk factors for disparities.

## METHODS

### Cohort

**FIGURE 1:**
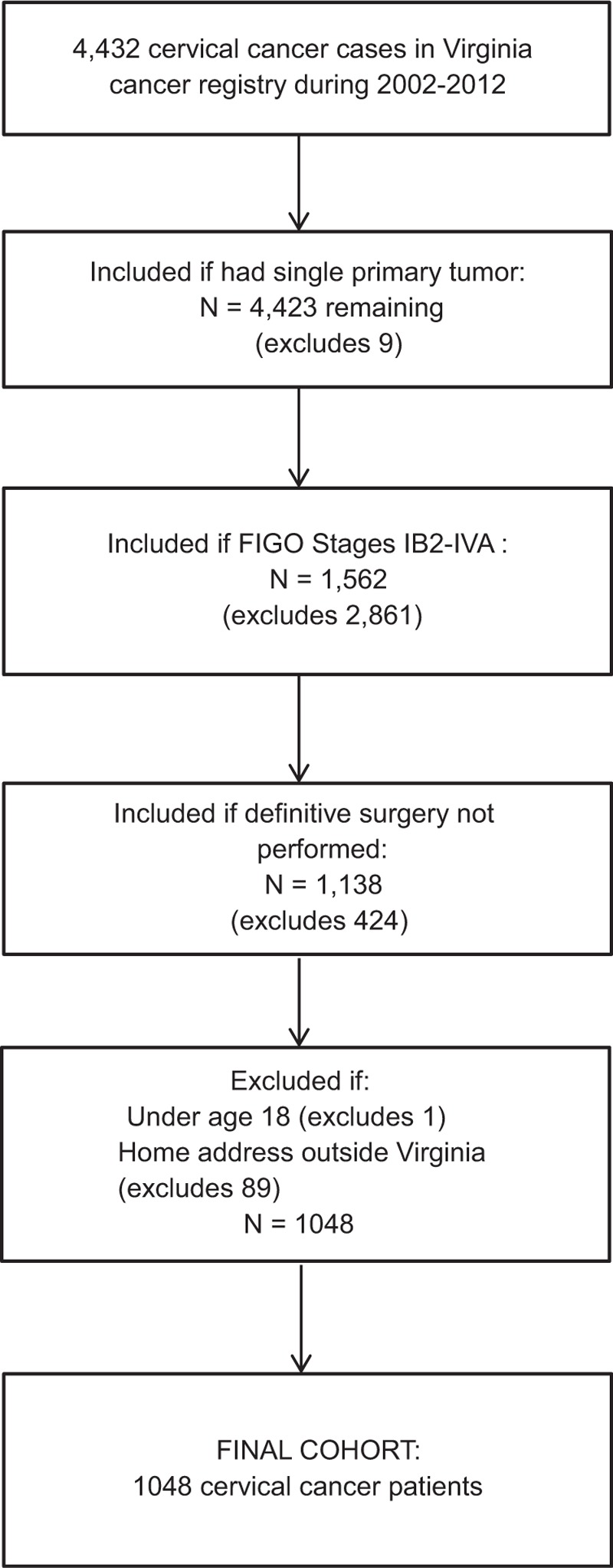
Selection strategy for the cohort in the current study, which is comprises female residents of Virginia who were diagnosed with cervical cancer during 2002 to 2012.

After receiving approval from the University of Virginia Institutional Review Board and the Virginia Department of Health, we used the state cancer registry to identify an observational cohort from among adult (ages 18 years and older) female residents of Virginia who were diagnosed with locally advanced cervical cancer during 2002 to 2012. The year 2002 is chosen to include patients treated in an era with contemporary BT and with concurrent CT established as standard of care (which occurred in 1999^6^). Cervical cancer diagnosis was defined based upon International Classification of Disease sites C530 to C539 and histology codes not in 9590–9992, 9050–9055, and 9140. Patients with International Federation of Gynecology and Obstetrics stages IB2-IVA tumor were included. Patients were excluded if they received definitive surgery, if there was a previous primary tumor, or if there were distant metastases (Figure 1).

### Predictors

For each subject, we recorded year of diagnosis, age at diagnosis, tumor staging information, race, insurance status, place of service, and home location. The following other regional factors were identified from the Area Health Resource File United States Department of Agriculture Rural-Urban Continuum Codes: metropolitan (1–3), urban nonmetropolitan (4–7), rural location (8–9), median income distribution (American Community Survey median income), annual poverty level as defined from the US Census Bureau SAIPE estimates (Small Area Income & Poverty), and percentage of patients with more than high school education. Facility identifiers were used to define characteristics of treatment facilities for each subject, including academic (versus nonacademic) centers based on American Medical Association records and volume of cervical cancer cases seen per year. For volume, facilities were categorized based on quartiles, where a high-volume facility was defined as the top quartile of annual cervical cancer cases. Study patients were linked to the highest volume facility where care was received, and therefore could have received treatment at more than one facility. Geo-coding was performed to identify travel and straight-line distances from patient's home location to the nearest academic or high-volume facility. Current presence of a high-dose rate (HDR) BT afterloader was identified based on records obtained from vendors, but data were not available to document presence of a HDR BT afterloader or sources for low-dose rate BT during the study period.

### Outcomes

Treatment outcomes included the presence of CT, presence of BT, and a quality score. Quality scores range from 0 to 3 defined as the sum of one point each for receipt of BT, CT, and EBRT treatments. Logistic regression models with random intercepts were used to assess the association between potential predictors shown in Table 1 and presence of CT/BT, with random intercept representing unmeasured effects of largest volume treatment facility. The xtlogit procedure in Stata 13 was used to fit the models. The analyses focused on CT and BT, rather than EBRT, as these are the modalities with previously reported trends towards declining utilization.^7^ For each modality (CT and BT) in Table 1, analysis of predictors of treatment receipt evaluated that specific treatment regardless of whether other modalities were also delivered.

**TABLE 1:**
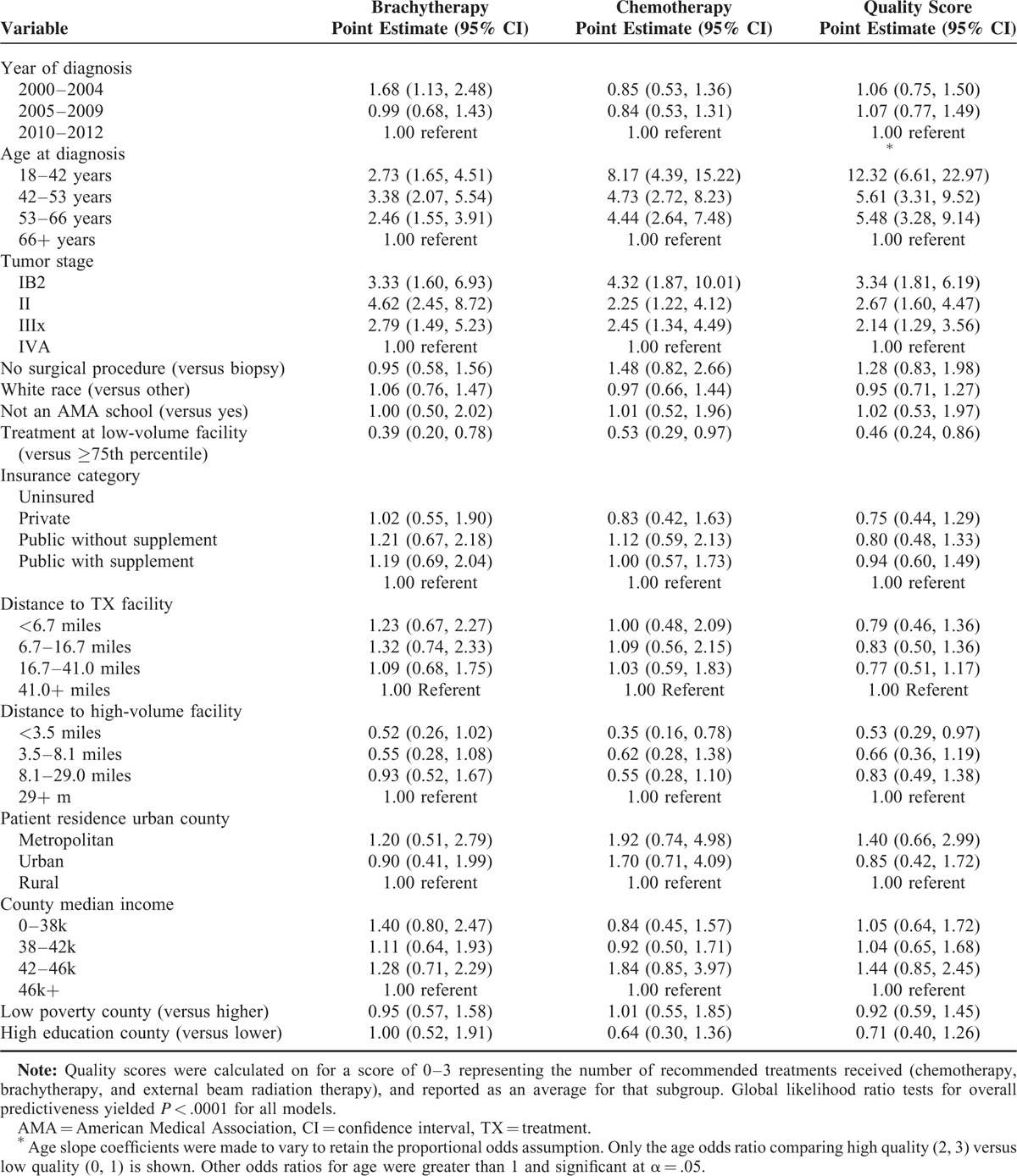
Predictors of Treatment Delivery

Since quality score was ordinal, a random-intercept partial proportional odds logistic regression fitted the data with quality score as the outcome and Table 1 predictors, allowing for variation in fixed effect coefficients corresponding to age in order to satisfy the proportional odds assumption. As previously, the random intercept was chosen to represent unmeasured effects of the largest volume treatment facility. The Stata module ‘Generalized linear latent and mixed model ’ with the method of adaptive quadratures was used to get the parameter estimates.

Survival outcomes included all cause survival and net cancer-specific survival (the probability of surviving cancer in the absences of other causes of death). Using the Stata procedure ‘stcox’, a Cox Proportional Hazards model was fit to assess the associations of survival with predictors shown in Table 2, including treatment quality, with the proportional hazards assumption being examined with the ‘phtest’ function in Stata. Mortality events were identified based on Virginia coroners’ data, with cases without evidence of death censored November 1, 2014. The cohort was restricted to those patients who survived for at least 6 months in order to minimize the effect of immortal time bias,^9^ which would bias survival in favor of the completely treated cases, as these cases cannot die between diagnosis and treatment initiation.

**TABLE 2:**
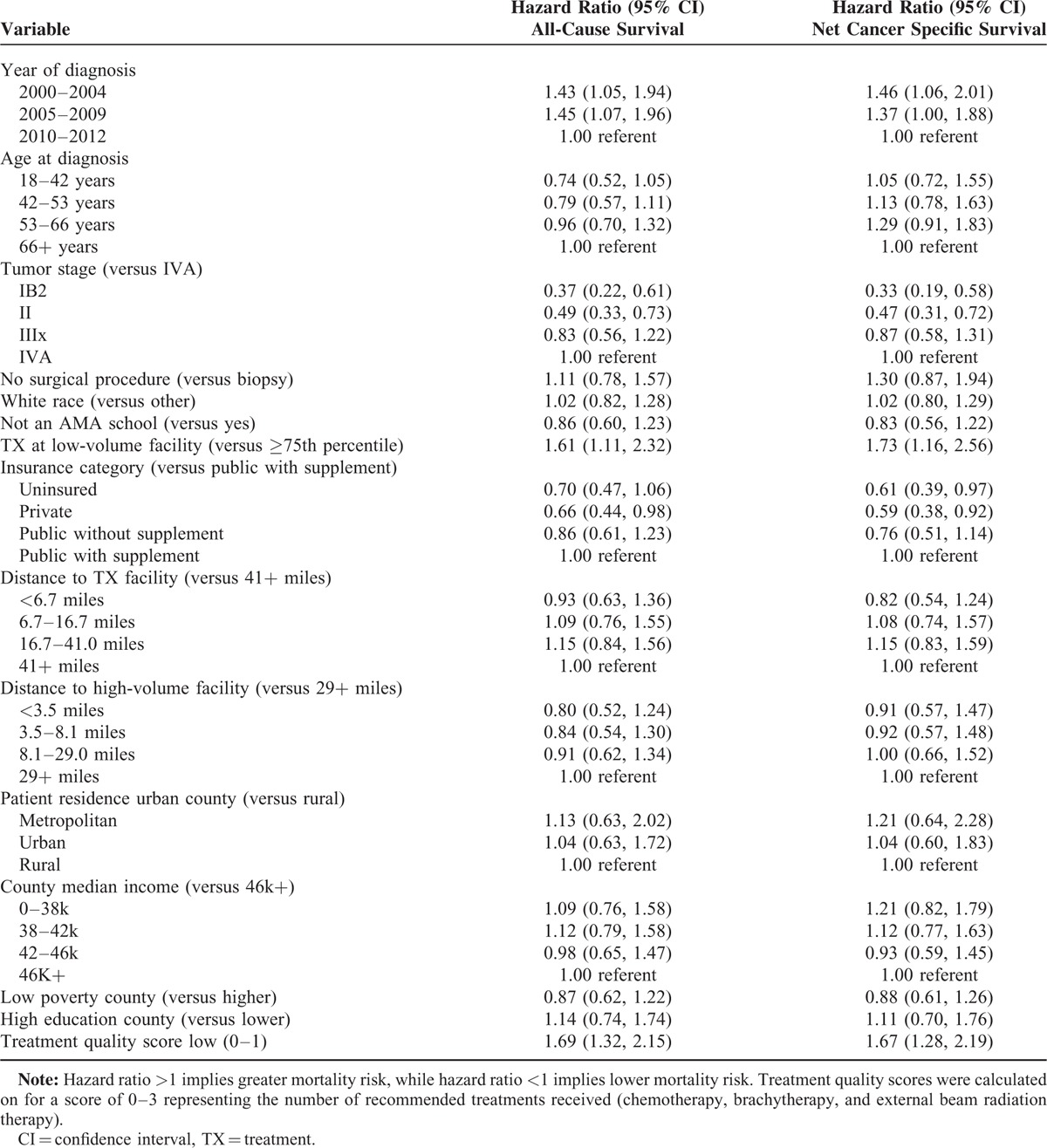
Cox Proportional Hazards Model for All-Cause Mortality

### Mediation Analysis

**FIGURE 2:**
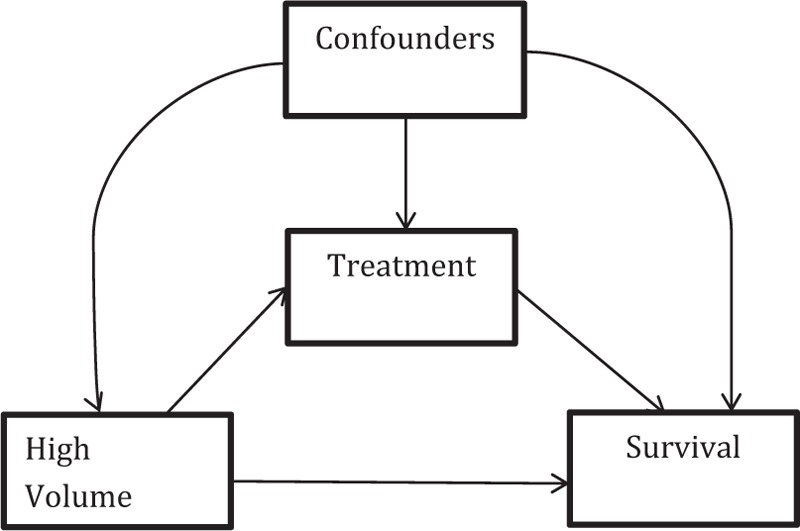
The direct acyclic graph assumed in the mediation analysis of survival. Chosen confounders included significant predictors of treatment and survival: year of diagnosis, age at diagnosis category, stage, and distance to nearest large facility category.

A mediation analysis method proposed by Lange et al^10^ based on potential outcome theory was performed to in order to estimate the natural direct effect and indirect effect of high-volume facility (L) on all-cause survival (S) where the effect is mediated by binary quality score (0–1 versus 2–3) (Q). The causal graph under which the mediation analysis can provide identified and unbiased estimates of the effects is shown in Figure 2. Furthermore, censoring is assumed to be noninformative.

Under the mediation model and assuming S satisfies the proportional hazards assumption, the total effect of L on S is given by TE = λʎ_L = 1_(t)/ʎ_L = 0_(t), where ʎ_L = 1_(t) is the hazard for high-volume facilities compared with ʎ_L = 0_(t), the hazard for low-volume facilities. The total effect can be decomposed into direct and indirect effects given by TE = (ʎ_L = 1Q (L = 1)_/ʎ_L = 0Q (L = 1)_) ×^ ^(ʎ_L = 0Q (L = 1)_/ʎ_L = 0Q (L = 0)_) (t), where the first term is the natural direct and second term is the indirect effect.^11^ The hazard rate ʎ_L = 0Q (L = 1)_(t) corresponds to the counterfactual survival curve observed for patients at low-volume facilities and with mediator Q set to the value, which would have been observed in high-volume facilities. Thus, the direct effect hazard ratio compares high- versus low-volume facility effects at a constant level of Q unaffected by volume.

We applied the method of Lange et al^10^ to obtain unbiased estimates and standard errors of the effects using a Marginal Structural Model, which can be obtained from a weighted Cox regression on a duplicated data set, where the regression includes adjustment for prior confounders C. The weights in the duplicates are the ratio of the conditional probabilities of the mediator (Q), P (Q = Q_obs_|L = 1 − L_obs_, C)/P(Q = Q_obs_|L = L_obs_, C), which were estimated in this study using logistic regression models. The SAS system v9.4 was used for this analysis.

Besides investigating mediation on large volume on survival, we also sought to investigate mediation of high-volume facilities on receipt of BT with the presence of BT source as mediator. The rationale of this secondary analysis was to assess whether the main pathway to receipt of BT by large volume facilities is through the simple presence of a HDR BT afterloader unit at the facility. In this case, we used the same mediation analysis method described upon, which can be extended to mediation models for which the outcome is dichotomous. The total effect odds ratio (OR) can be decomposed similarly as the total effect hazard ratio.

## RESULTS

A total of 1048 patients were identified who met study inclusion criteria (Figure 1). Among this cohort, a total of 54.0% received BT, 77.1% received CT, and 76.6% received EBRT. Only 33.1% of subjects received all three treatments (EBRT, BT, and CT). The other common treatment combinations, in descending order of frequency included EBRT + CT (28.6%), BT + CT (14.4%), and EBRT alone (11.4%). Quality scores of 0, 1, 2, and 3 were observed among 4.7%, 15.6%, 46.5%, and 33.1% of patients, respectively.

Characteristics of the cohort are shown in Table 3 . Most patients were stage IIx (39.3%) and IIIx (41.7%), had no surgical procedures (91%), were white (67.2%), received treatment at a high-volume facility (87.2%), American Medical Association school facility (85.5%), had private insurance (36.8%), lived in a metropolitan county (80%), and lived in highest income bracket (46K+, 55.7%), low poverty (83%) and higher education counties (86.6). Mean age at diagnosis was 55 years (SD = 15.8), with minimum of 18 years and maximum of 96 years. Mean driving distance to largest volume treatment facility was 26 miles (median, 14 miles). Mean straight-line distance to nearest high-volume facility was 16 miles (median, 7 miles).

**TABLE 3:**
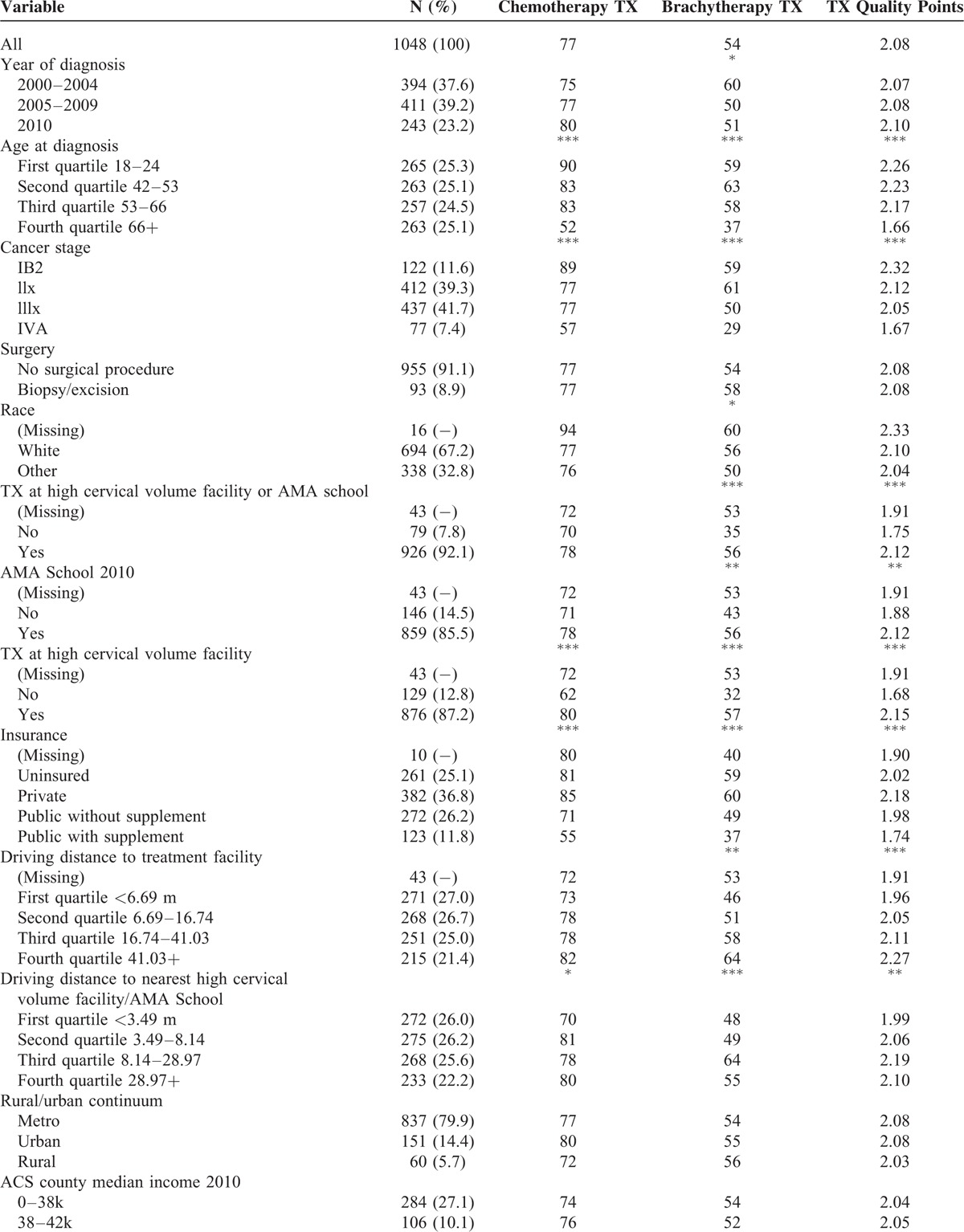
Characteristics of the Cohort

**TABLE 3 (Continued):**
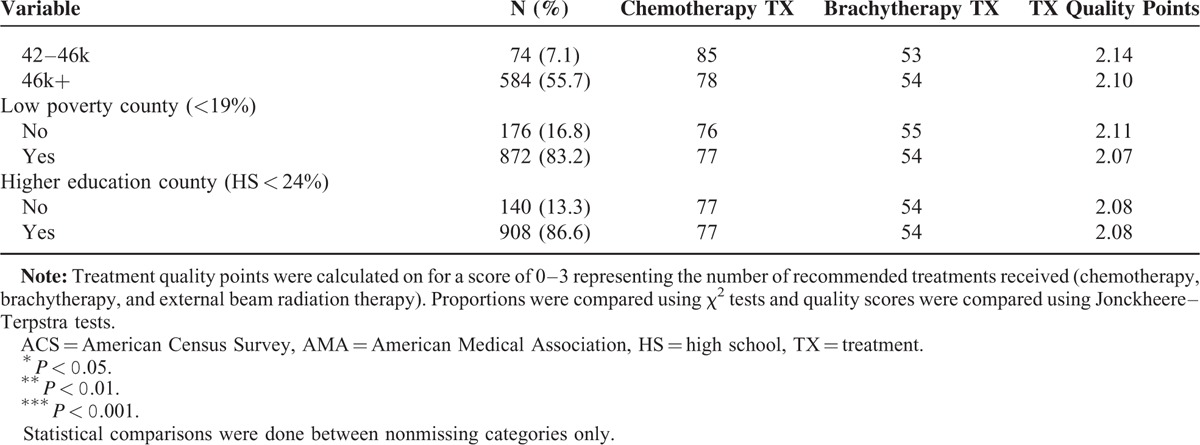
Characteristics of the Cohort

### Predictors of Treatments Delivered and Quality Score

The results of the multivariable analyses of predictors for receipt of BT and CT, and for quality score, are shown in Table 1. Increased probability of BT delivery was associated with earlier era of diagnosis [2000–2004 versus 2010–2012 OR = 1.68, 95% confidence interval (CI): 1.13, 2.48], younger age versus 66+ years at diagnosis (18–42 OR = 2.73, CI: 1.65, 4.51; 42–53 OR = 3.38, CI: 2.07, 5.54; 53–66 OR = 2.46, CI: 1.55, 3.91), and lower tumor stages versus IVA (IB2 OR = 3.31, CI: 1.60, 6.93; II OR = 4.62, CI: 2.45, 8.72; IIIx OR = 2.79, CI: 1.49, 5.23), while decreased probability of BT delivery was associated with treatment at a high-volume facility (OR = 0.39, CI: 0.20, 0.78) (Table 1). Increased probability of CT delivery was associated with younger age versus 66+ years (18–42 OR = 8.17, CI: 4.39, 15.22; 42–53 OR = 4.73, CI: 2.72, 8.23; 53–66 OR = 4.44, CI: 2.64, 7.48), and lower tumor stages versus IVA (IB2 OR = 4.32, CI: 1.87, 10.01; II OR = 2.25, CI: 1.22, 4.12; IIIx OR = 2.45, CI: 1.34, 4.49), while treatment at a low-volume facility is associated with decreased probability of CT delivery (OR 0.53, CI: 0.29,0.97) (Table 1). Predictors of higher quality score included younger age group versus than 66+ years at diagnosis (18–42 OR = 12.32, CI: 6.61, 22.97; 42–53 OR = 5.61, CI: 3.31, 9.52; 53–66 OR = 5.48, CI: 3.28, 9.14), and lower tumor stages versus IVA (IB2 OR = 3.34, CI: 1.81, 6.19; II OR = 2.67, CI: 1.60, 4.47; IIIx OR = 2.14, CI: 1.29, 3.56), while treatment at a low-volume facility was associated with lower quality score (OR 0.46, CI: 0.24, 0.86) (Table 1). Home location less than 3.5 miles from a high-volume facility was associated with an unexpectedly lower likelihood of receiving CT and lower quality score (Table 1). In order to examine whether the effect of facility volume on outcome varied by stage, interaction terms were added to each of the three models and overall interaction between volume and stage effect was tested. No statistically significant interaction (*P* < .05) was found.

### Survival

A total of 887 subjects were included after removing patients who survived for less than 6 months after diagnosis and who had incomplete data. Mean follow-up time for the patients was 5.2 years, with a median of 3.8 years. The results of the Cox proportional hazards model are displayed in Table 2. Predictors of increased risk of mortality included earlier year of diagnosis, higher tumor stage, treatment at a lower volume facility, and treatment quality score of 0 to 1 (versus 2–3). The estimated hazard ratio (HR) for survival for low versus high facility volume was HR = 1.61 (CI: 1.11, 2.33), suggesting high-volume facilities are likelier to be protective, and HR = 1.69 (CI: 1.32, 2.16) for quality score of 0–1 (versus 2–3), suggesting lack of recommended treatments is associated with lower survival rates (Table 2). At the end of 2 years, survival adjusted for other covariates was 76% for patients in high-volume facilities who received high treatment quality (scores 2–3), 65% for high-volume facility and low treatment quality (scores 0–1), 64% for low-volume and high treatment quality, and 49% for low-volume and low treatment quality. At the end of 5 years, survival rates were 62%, 48%, 46%, and 30%, respectively for these categories. When the outcome was net cancer-specific survival, the hazard ratio for low versus high facility volume was stronger, HR = 1.73 (CI: 1.16, 2.56), and comparable in magnitude with overall survival for the low-quality score 0–1 versus 2–3 HR = 1.67 (CI: 1.28, 2.19) (Table 2).

## RESULTS FOR MEDIATION ANALYSIS

### Effect of High Volume on Observed All-Cause Survival mediated by Treatment

The list of predictors of survival used to control for confounding is found in Table 2. Race and insurance status were excluded to preserve sample size from missing data and because they were determined to be weak predictors in multivariable regression models (*P* > 0.15). The natural direct effect within strata of the potential confounders of high-volume facility on survival was given by the hazard ratio HR = 0.68 (CI: 0.53, 0.87), in a protective direction. The indirect effect was HR = 0.96 (CI: 0.84, 1.10), in a protective (but nonsignificant) direction. The total effect was given by HR = 0.68^∗^0.96 = 0.65, suggesting that receipt of complete treatment (EBRT + CT + BT) is not the sole mediator of the relationship between high-volume facility and survival and that other pathways are present by which treatment at large volume facilities may improve survival.

### Mediation of Effect of High-Volume Facility on Brachytherapy Delivery by Presence of Brachytherapy Source

Because the available data for presence of BT sources is current (2014), and does not necessarily represent resources during the entire study period, this mediation analysis was performed only on patients diagnosed after 2010, for a total of 236 patients. The list of potential confounders of BT and BT source consisted of all predictors in Table 1, again with the exception of race and insurance. Within this strata of covariates, the OR for the natural direct effect was OR = 2.16 (CI: 1.56, 2.98) such that high-volume facilities are more likely to provide BT and the OR for the indirect effect was OR = 1.42 (CI: 0.36, 5.56), yielding a total effect of OR = 2.16^∗^1.42 = 3.07. Standardizing the probability of BT delivery using the covariate mix in the sample, we find the mediation model predicts 52% of patients in high-volume facilities receive BT treatment, compared with 30% for low-volume facilities for patients diagnosed after 2010. Since the indirect effect is not significant, we cannot assess the extent of mediation. However, the total marginal effect can be hypothetically decomposed additively into direct and indirect effects as (52%–45%) +  (45%–30%), where 45% is the counterfactual rate of BT delivery in low-volume facilities with presence/absence of BT source chosen according to the hypothetical case in which the facilities would have been of high volume. The proportion explained by presence of BT source is then as high as 68%.

## DISCUSSION

In an observational cohort of female Virginia residents diagnosed with locally advanced cervical cancer from 2002 to 2012, we found that only one-third of subjects received all components of recommended treatment (EBRT, BT, and CT). Treatment at a medical facility with a high annual volume of cervical cancer patients was associated with higher rates of receipt of quality treatment. Higher quality score, representing a summary index of the amount of compliance with recommended treatment modalities, and treatment at a high-volume facility were associated with higher survival rates. Together, these findings highlight a low rate of utilization of the core modalities that comprise quality care for locally advanced cervical cancer and demonstrate improved treatment quality and survival for patients who are treated at high-volume medical centers.

The low rates of BT (54.5%) and CT (77.3%) delivery observed in our study are consistent with previous reports that evaluated national databases. Smith et al reviewed an employment-based health claims database and found that only 44% of a cohort of insured cervical cancer patients met all 3 benchmarks for quality care.^12^ A national survey of radiation therapy facilities across the United States revealed a high rate of noncompliance with established criteria for high-quality care, including delivery of concurrent CT and BT, particularly among nonacademic facilities.^4^ Noncompliance with BT and CT is an important problem to address, as it has a strong impact on survival. In our study, we found that poor treatment quality was a strong predictor of all-cause mortality as well as cancer specific mortality. Other studies have shown that omission of BT is associated with a corresponding increase in mortality.^4,13^ Han et al^7^ observed an improvement in survival with BT, in adjusted analyses using propensity score matching methods. A prior analysis of the National Cancer Database has shown that the survival detriment from omission of BT was strong than that of omission of CT.^14^ These observations regarding the trend towards declining rates of BT and CT utilization, and the major associated survival detriments, provided our rationale for focusing our analyses on CT and BT delivery in the current study. Improvement of the rates of compliance with quality treatment delivery for locally advanced cervical cancer is an important goal, as much could be gained in terms of improving clinical outcomes by simply improving the delivery of quality, standard definitive treatment for locally advanced cervical cancer.^15^

There seems to be a trend towards decreasing utilization of BT in recent years. In our analysis, BT delivery was more likely in 2000 to 2004 than in later years. Han et al reviewed the SEER database and observed a decline in BT utilization from 83% in 1988 to 58% in 2009. Interestingly, the sharp decline of 23% in 2003 (down to 43%) suggested the potential influence of reliance upon newer EBRT approaches as a replacement for BT.^7^ Gill et al identified a trend towards decreasing utilization of BT over time in the National Cancer Database, with a 10% decline in utilization from 2004 to 2011, with an associated decrease in survival rates.^14^ Decreased utilization of BT was associated with later year of diagnosis, lower volume facility, and facility type.^14^ Determining the primary explanations for the low and decreasing utilization rate of BT is beyond the scope of this study.

In the current study, treatment at a high-volume treatment facility was a significant predictor of a higher quality score, and was a significant predictor of survival, findings that are consistent with the existing medical literature. Higher volume centers have been previously reported to be associated with improved survival after treatment for cervical cancer, as well as increased likelihood of receiving CT and BT and shorter duration of radiation therapy.^13^ A review of the National Cancer Data Base showed that patients who were treated at higher volume facilities were more likely to receive concurrent CT with radiation therapy, compared with radiation therapy alone.^16^ Based upon our observations in the current study, it is the receipt of care at a high-volume center that is associated with quality score and survival outcomes, and not necessarily the distance from a patient's home to a high-volume center. Along these lines, a previously reported evaluation of patients referred to the University of Kentucky demonstrated that residence and location of primary treatment center were not predictors of cervical cancer survival when care was overseen by a tertiary care center.^17^ Based on these observations, developing formal interventions to help patients receive care at higher volume facilities may help address the disparities observed in our review of patients in Virginia. The finding of undertreatment of patients living within less than 3.5 miles of a high-volume center otherwise associated with better delivery of guideline concordant care raises concerns about potential lack of access of those living within urban core areas, such as due to poverty or insurance type. Further research is needed to identify the referral and access patterns of those who receive incomplete care for cervical cancer so that high-risk patients and populations can be included in cancer center outreach efforts.

Our study has some important limitations that must be considered when making conclusions regarding the observed results. First, the data available lacks important details regarding the radiation therapy, such as radiation dose and treatment duration, which may influence outcomes. In addition, it is possible that the receipt of BT, EBRT, and CT may be unascertained in this dataset, since patients who reside along the border of the state may receive some of their care elsewhere. However, the fact that our findings were consistent with the available evidence using national databases suggests that this would not have influenced our findings. Furthermore, we did not notice a trend toward decreased quality score at the state borders. Another significant limitation is the lack of information regarding pre-existing medical comorbidities or the extent of supportive care received during treatment, as these factors could confound the mortality analysis. Finally, we were not able to determine whether the availability of specialty providers, such as gynecological oncologists or radiation oncologists, or the presence of equipment, such as a BT sources or infusion centers, contributed to the observed effects for high-volume facilities, as provider data were not available for the facilities and the status of BT source presence was not known throughout the study period.

## CONCLUSIONS

In a cohort of female Virginia residents with locally advanced cervical cancer, we observed a low rate of compliance with basic components of quality care and a strong effect of facility volume on receipt of quality treatment and survival. Since high-volume centers are located mostly in urban and heavily populated areas of the state, our findings demonstrate an important disparity for cervical cancer patients with potential etiologies that may include geography, socioeconomic and organizational barriers to receiving high-quality care. Further research is warranted to better understand the low delivery rates of quality therapy. Potential interventions should be developed to improve access to high-volume treatment centers or to improve overall access to CT, BT, and EBRT through Virginia, as such efforts may deliver a substantial survival benefit for patients with locally advanced cervical cancer.
